# Randomized controlled trial on the effect of the peanut ball devices on the duration of active labor, labor pain and comfort

**DOI:** 10.1186/s12884-026-08720-2

**Published:** 2026-02-17

**Authors:** Elif Hoyladı, Yeliz Yıldırım Varışoğlu

**Affiliations:** 1 Institute of Health Science, Department of Midwifery, Istanbul Medipol University, Istanbul, Turkey; 2 Faculty of Nursing, Istanbul University, Istanbul, Turkey

**Keywords:** Peanut ball, Birth ball, Birth comfort, Labor pain, Length of childbirth

## Abstract

**Purpose:**

To evaluate the effect of peanut ball usage on labor pain birth comfort, and length of childbirth among pregnant women during childbirth.

**Methods:**

This randomized controlled trial included both primiparous groups, each of which was divided into intervention and control groups. 102 pregnant women were enrolled and randomized into the peanut ball group (PG: 51) and control groups (CG: 51). Experimental-group women used peanut balls during labor of active and transition phase. After the cervical dilatation reached 4 cm, the pregnant women in the PG performed peanut ball exercises, adhering to the peanut ball guide created by the researcher. Control-group women standard midwifery care for the same period as the experimental group. The Visual Analog Scale (VAS), the Birth Comfort Scale (BCS), and the Partograph were used to measure outcomes.

**Results:**

The process of dilation, effacement, and descent of the fetal head showed statistically significant differences between both groups after intervention (p<0.05). However, the length of childbirth was similar between both groups. The birth pain scores of the pregnant women in the PG were significantly lower and increased physical and psychospiritual comfort at birth (p<0.05).

**Conclusion:**

It was determined that the use of peanut ball by pregnant women during labor does not affect the length of childbirth, reduce labor pain, and increase physical and psychospiritual comfort.

**Trial registration:**

The study was retrospectively registered in National Library of Medicine Clinical Trials Registry (Date: 12.16.2023 Ref: NCT06200688).

**Supplementary Information:**

The online version contains supplementary material available at 10.1186/s12884-026-08720-2.

## Highlights

Peanut ball reduces labor pain during the active phase, transition phase, and second stage of labor.

Peanut ball application increased the physical and psychospiritual comfort level of birth.

The findings obtained from this study emphasize that the peanut ball is an alternative method that can be easily used during labor to reduce labor pain and have positive labor.

**Supplementary Information:**

The online version contains supplementary material available at 10.1186/s12884-026-08720-2.

## Statement of significance

*Problem or Issue*: Birth Pain and Comfort

*What is Already Known*: More studies should be performed to determine the benefits of birth ball exercise in labor.

*What this Paper Adds*: Penut ball could significantly reduce the birth pain response and increase physical and psychospiritual comfort in the labor process.

## Introduction

Historically, women and family members have been under the supervision of traditional birth attendants or midwives. Nowadays birth has become a medical event and has affected the mode of delivery [1–3].

Previous studies have emphasized that movement freedom and frequent position changes during birth provided many benefits to women in labor and could help to support birth physiology [1, 4–7].

The use of birth balls and peanut balls is a nonpharmacological method that is used for pain management at birth. Research suggests that upright positioning, such as squatting, during labor may widen the pelvic outlet and improve the progress of labor, shortening the length of childbirth, and reducing cesarean section rates [5–12].

This study aimed to determine the effect of a peanut ball, which is used in labor, on labor time, labor pain, and birth comfort. In this way, the aim was to reduce birth pain and increase birth comfort to increase the rate of vaginal birth, improve maternal-fetal birth outcomes, and improve women’s birth experiences.

The hypotheses of this study were as follows:

- H11: The labor pain of pregnant women who use a peanut ball is different from those who do not.
- H12: The birth comfort of pregnant women who use a peanut ball is different from those who do not.
- H13: the time from the active phase to the completion of the transition phase of pregnant women who use a peanut ball is different from those who do not use it.

## Methods

### Study design

**Fig. 1:**
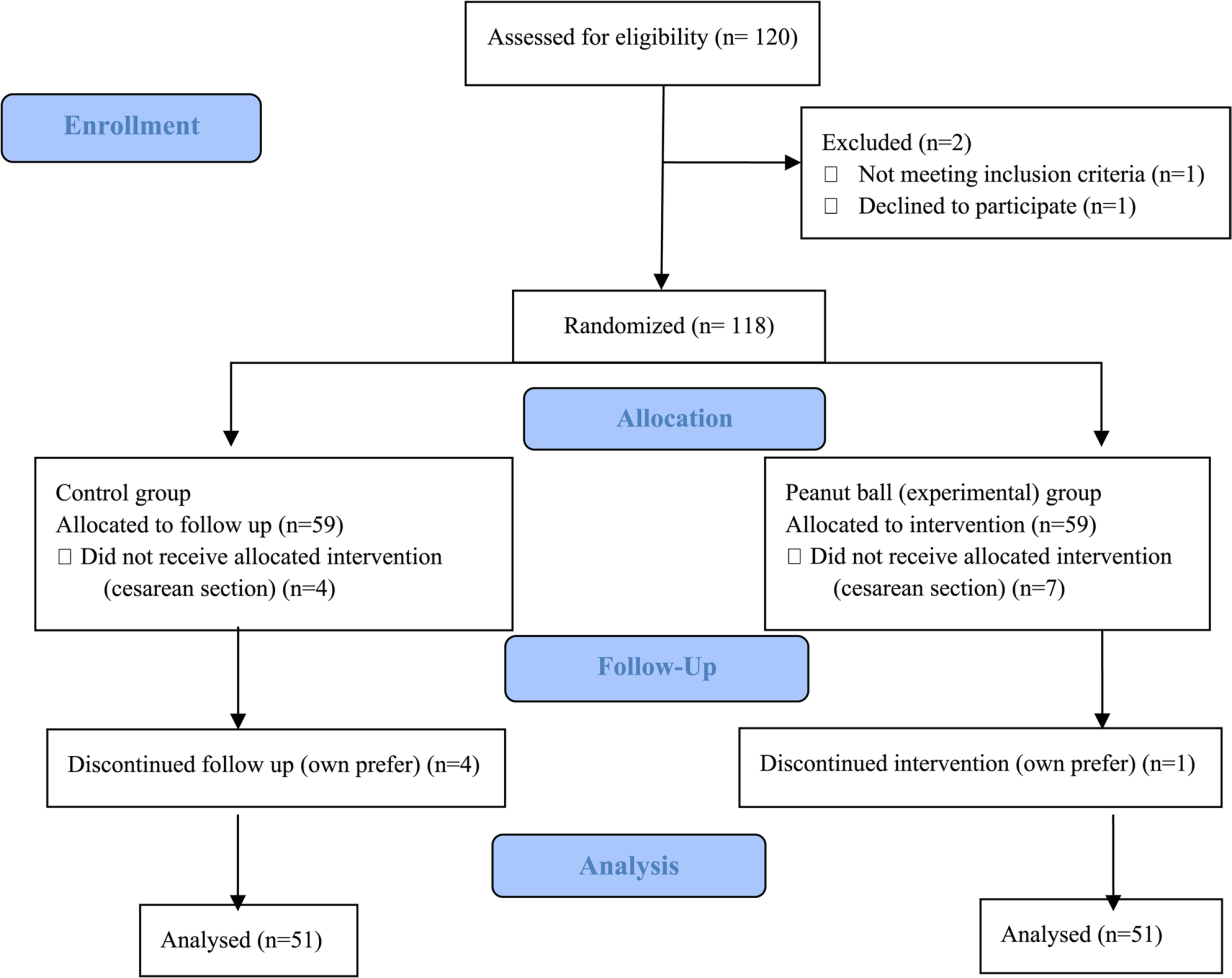
The process of the study is based on the CONSORT flow diagram

This study employed a randomized, controlled, and single-blind design, adhering to the guidelines of the Consolidated Standards of Reporting Trials (CONSORT) checklist. A total of 120 pregnant women were randomized into two groups: the intervention group (peanut ball group) and the control group. The flowchart summarizing the participant recruitment, allocation, and analysis is provided in Fig. 1.

### Study participants

The study was conducted in the delivery rooms of a Training and Research Hospital in Istanbul, Turkey. Pregnant women who volunteered to participate were evaluated based on the following inclusion criteria:

- Gestational age between 37 and 42 weeks,
- Age ≥ 18 years,
- Singleton pregnancy,
- Cervical dilatation of 4 cm,
- Being primiparous,
- No complications contraindicating vaginal delivery,
- Not having received epidural anesthesia or analgesia,
- Ability to communicate in Turkish.

Exclusion criteria included cesarean delivery, administration of analgesia, or voluntary withdrawal from the study for personal reasons. Out of 118 eligible participants, 11 were excluded due to cesarean section, and 5 withdrew from the intervention. As a result, 102 pregnant women completed the study, with 51 participants in each group.

### Sample size determination

The sample size was calculated based on labor pain scores reported in the study by Delgado-García et al. (2011) [13]. Using the G*Power 3.1.9.2 software (Franz Faul, Universität Kiel, Germany) with a 95% confidence interval and 80% power, the required minimum sample size was determined to be 38 participants per group. To account for potential attrition, the target enrollment was set at 60 participants per group. Ultimately, 51 participants in each group completed the study, allowing for an estimated attrition rate of 30%.

### Randomization

A computer-based randomization process was used to allocate participants into the intervention or control groups. Random numbers were generated using the Randomizer.org website. The allocation sequence was created before the study began and kept concealed from the investigators responsible for participant recruitment to minimize selection bias. Pregnant women were assigned to their groups sequentially based on their order of enrollment and the pre-determined randomization list.

Blinding was maintained by ensuring that the participants were unaware of their group assignments. Although the researchers administering the interventions were aware of group allocation, the outcomes were assessed by independent evaluators blinded to the group assignments.

## Materials

### Peanut ball

A peanut ball is shaped like a peanut shell, with a narrower middle circumference compared to the ends. To facilitate optimal positioning for labor progression, the peanut ball can be placed between the legs of a laboring woman who is confined to bed. This positioning aims to increase pelvic diameter, providing more space for fetal descent. Although the peanut ball is widely used in labor and delivery units, there is limited research supporting its effectiveness in improving labor outcomes or managing labor pain. This midwife-led study was designed to evaluate the effectiveness of the peanut ball in reducing the length of labor, alleviating labor pain, and improving childbirth comfort in a randomized controlled trial comparing women who used the peanut ball to those who did not.

## Interventions

Participants in the intervention group performed peanut ball exercises during labor in addition to receiving routine midwifery care. These exercises were standardized to ensure consistency in implementation. The control group received only routine midwifery care without any additional interventions. All participants were monitored throughout labor, and data were collected systematically to ensure accuracy and reliability methods.

After obtaining informed consent, participants were assigned to groups based on their order of acceptance. The study’s purpose was explained to all participants, and they were assured that the data would remain anonymous, and confidentiality principles would be upheld.

Peanut Ball Group (PG): Participants received detailed information about the peanut ball and its proper usage. A total of five positions were demonstrated. Once cervical dilation reached 6 cm, the peanut ball was introduced. Participants were encouraged to change positions every 30–45 min. The peanut ball was utilized during the active and transition phases of labor, ensuring that participants felt comfortable selecting their positions.

Control Group (CG): Participants in the control group received no additional intervention beyond routine clinical practices. These women were generally observed to spend more time lying down and undergoing electronic fetal monitoring until delivery.

### Measurements

The data of the study were collected between July 2021 and July 2022 in a Training and Research hospital in Istanbul. The following tools were used in this study:

Pregnancy Information Form: This form, developed by the researchers based on a review of the literature, consisted of 18 questions to assess the sociodemographic and obstetric characteristics of the participants.

Partograph: The partograph included details such as the participant's name, gravidity, protocol number, date, time of admission, and labor progression. Cervical dilation, fetal head station, and the frequency and duration of uterine contractions were monitored and recorded every 10 minutes. Fetal heart rate, amniotic membrane status, and amniotic fluid color were evaluated and recorded every 30 minutes. Labor duration was measured in minutes, and the frequency and duration of contractions were expressed as averages in seconds.

**Fig. 2:**
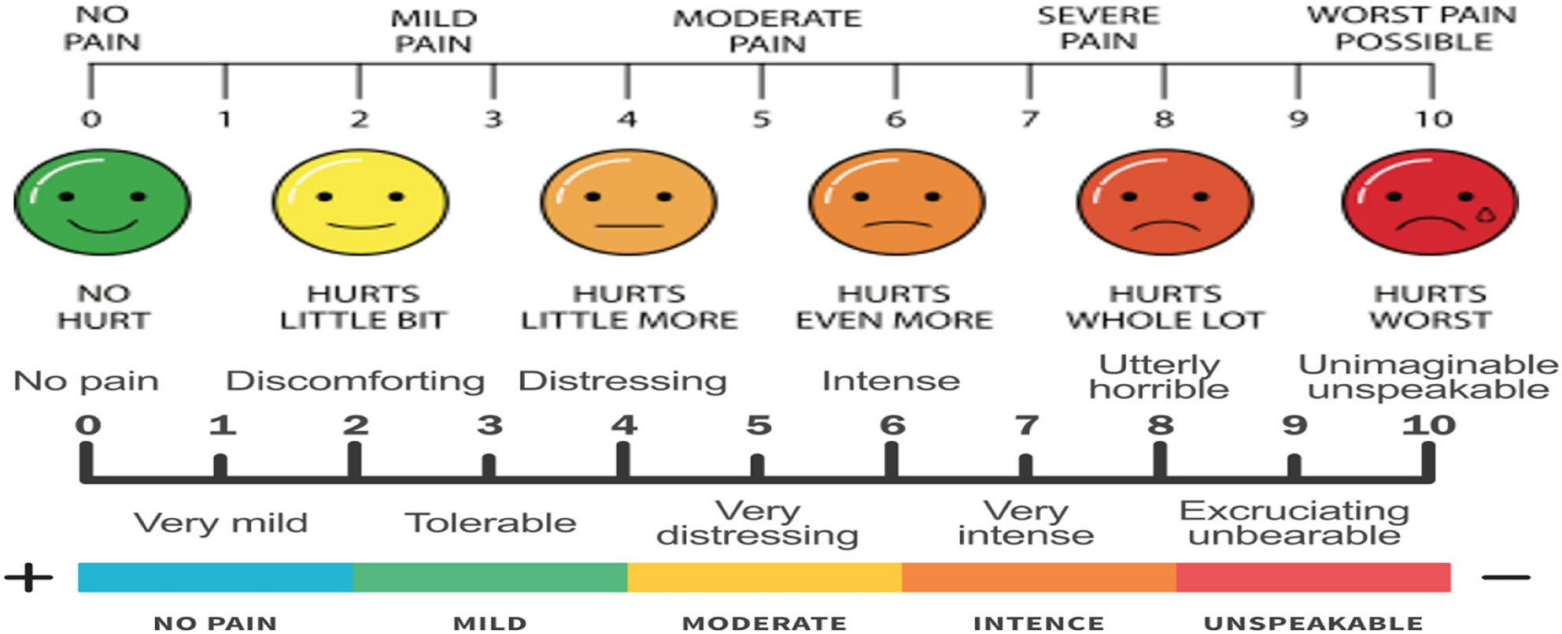
Visual analog scale

Visual Analog Scale (VAS): The VAS is a widely used, simple, and effective tool to measure pain intensity (Fig. 2).

Birth Comfort Scale: Originally developed as the "Childbirth Comfort Questionnaire" by Kerri Durnell Schuiling and based on Kolcaba's comfort theory, the Turkish validity and reliability study was conducted by Potur et al. (2015). The scale used in this research included 9 items, rated on a 5-point Likert scale (1 = strongly disagree, 5 = strongly agree). The total score ranged from 9 to 45, with higher scores indicating greater comfort. Each item assessed specific dimensions of comfort, such as physical, environmental, psychosocial, and spiritual factors [14].

### Statistical analysis

Data were analyzed using the Statistical Package for the Social Sciences (IBM SPSS) version 22.0. The Shapiro-Wilk test was used to assess normality (*p* ≥ 0.05). For normally distributed data, a paired t-test was used for within-group comparisons, and an independent t-test was used for between-group comparisons (e.g., VAS). Categorical variables were analyzed using chi-square or Fisher’s exact tests. Quantitative continuous data were compared between two groups using independent t-tests, while dependent group t-tests were applied for within-group comparisons. Statistical significance was set at *p* < 0.05.

### Ethical considerations

This study adhered to ethical principles for clinical research involving human participants and was approved by the Ethics Committee of Istanbul Medipol University (decision dated 21.06.2021, number 682). The trial was registered at clinicaltrials.gov (registration number: NCT06200688). Permission was obtained from the Provincial Health Directorate of the affiliated state hospital, and written informed consent was obtained from all participants. The study was conducted following the principles outlined in the Declaration of Helsinki.

## Results

Comparisons of sociodemographic characteristics, economic characteristics, obstetric characteristics, and preparation for birth are presented per group in Table 1. Comparisons of sociodemographic characteristics, economic characteristics, obstetric characteristics, and preparation for birth were found to be similar between the groups (*p* > 0 0.05).

**Table 1:** Characteristics of participant

		Peanutball Group		Control Group		
		Mean	sd	Mean	sd	test
Maternal age		25.47	4.38	25.06	3.78	t: 0.5 p:0.613
Geastational age		39.16	1.16	39.47	1.22	t: -1.332 p: 0.186
		n	%	n	%	
Educational status	Primary school	17	33.3	16	31.4	X^2^= 0.06 *p* = 0.970
	High school	16	31.4	17	33.3	
	University	18	35.3	18	35.3	
Working status	Employed	10	19.6	10	19.6	X^2^= 0.00 *p* = 0.598
	Unemployed	41	80.4	41	80.4	
Income rate	High income	3	5.9	4	7.8	X^2^= 0.40 *p* = 0.816
	Middle income	31	60.8	28	54.9	
	Low income	17	33.3	19	37.3	
Family type	Nuclear family	45	88.2	42	82.4	X^2^= 0.70 *p* = 0.289
	Extended family	6	11.8	9	17.6	
Marital status	Married	50	98.0	47	92.2	X^2^= 1.89 *p* = 0.181
	Single	1	2.0	4	7.8	
Prenatal childbirth preparation course	No	44	86.3	41	80.4	X^2^= 0.63 *p* = 0.298
	Yes	7	13.7	10	19.6	

Data are expressed as mean (SD) or count (%)

The progress of the labor process, cervical dilatation, effacement, descent of the fetal head, and length of the active and transitional phases of labor are shown in Table 2. Women in the PG completed the first stage of the mean of 130.6 ± 76.5 min, the mean of 134.5 ± 92.1 min in the CG. The second stage of labor was determined to be 52.9 ± 4.8 min in the PG and 54.2 ± 41.2 min in the CG. Although labor of the length of stage I and II of pregnant women in the PG was shorter than that of pregnant women in the control group, the difference was not statistically significant (*p* > 0.05).

**Table 2:** Distribution of participants’ cervical dilatation, effacement, fetal head descent, and length of active and transitional phase

		Peanutball Group (*n*:51)		Control Group (*n* = 51)			
		Mean	SD	Mean	SD	t^a^	*p*
Active phase dilatation (cm)	Pre-intervention	5.9	0.3	6.1	0.2	-0.405	0.687
	After intervention	7.9	0.8	6.1	0.2	16.428	< 0.001
t^b^ /p: -17.832/ *p*<0.001							
Active phase effasman (%)	Before intervention	71.6	8.3	67.3	7.8	2.704	0.008
	After intervention	78.8	6.5	67.3	7.8	8.125	< 0.001
t^b^ /p: 7.783/ *p*<0.001							
Active phase fetal head level	Before intervention	-1.8	0.6	-2.2	0.8	2.377	0.019
	After intervention	-0.7	0.9	-2.2	0.8	9.005	< 0.001
t^b^ /p: -15.327/ *p*<0.001							
Transition phase dilatation (cm)	Before intervention	8.2	0.5	8.2	0.3	0.4	0.706
	After intervention	9.8	0.4	8.2	0.3	23.9	< 0.001
t^b^ /p:-18.667/ *p*<0.001							
Transition phase effacement (%)	Before intervention	79.2	4.9	74.8	6.1	3.9	< 0.001
	After intervention	96.6	6.9	74.8	6.1	16.7	< 0.001
t^b^ /p: -15.3/ *p*<0.001							
Transition phase fetal head level	Before intervention	-0.5	0.8	-1.1	0.9	3.5	< 0.001
	After intervention	0.8	1.1	-1.1	0.9	9.6	< 0.001
t^b^ /p: -11.287/ *p*<0.001							
Length of Active phase		79.3	52.6	80.9	60.5	-0.148	0.882
Length of Transition phase		51.3	33.3	53.5	45.4	-0.286	0.775
Length of Stage 1		130.6	76.5	134.5	92.1	-0.234	0.816
Length of Stage 2		52.8	45.8	54.2	41.2	-0.139	0.89

*SD* Standard Deviation

t^a^ Independent Groups t-test; t^b^ Dependent Groups t-test

There was no significant difference between the VAS scores of the CG in the latent phase (*p*>0.05), active phase, or transition phase. The VAS score was significantly lower (*p* < 0.05) in the PG. When VAS measurements pre- and post-peanut ball application were compared in the PG, the active phase, and transition phase VAS score was found to be statistically significant (*p* < 0.05) (Table 3).

**Table 3:** Distribution of participants’ active phase, transition phase, and second stage VAS scores of labors

	Peanut ball Group (*n* = 51)		Control Group (*n* = 51)									
	Mean	SD		Mean		SD			t			*p*
Latent phase VAS	5.6	1.4		5.78		1.2			-0.757			0.451
Active phase pre-intervention VAS	7.1	1.2		7.69		0.7			-2.897			0.005
Active phase after intervention VAS	6.2	1.1		7.69		0.7			-8.067			< 0.001
	t^b^ : 8.267											
	*p*<0.001											
Transition phase pre-intervention VAS	8.2	0.8		8.8		0.7			-4.206			< 0.001
Transition phase after intervention VAS	7.2	1.1		8.8		0.7			-9.333			< 0.001
	t^b^ : 9.716											
	*p*<0.001											
	*p*<0.001											
Physically comfort	15.2	1.5		13.8		2.5			3.352			< 0.001
Phsicospirituel comfort	5.2	0.9		3.8		1.3			6.047			< 0.001
Environmental comfort	8.4	1.4		10.9		2.1			-6.987			< 0.001
Total	28.8	2.3		28.6		2.8			0.576			0.566

*SD* Standard Deviation

t^a^ Independent Groups t-test; t^b^ Dependent Groups t-test

The Peanut ball positions and opinions of the participants during the labor process are shown in Table 4. Thirty-six of the pregnant women were in the right/left side position, 3 were in the semi-sitting position, and 12 were in the ball-sitting position in the active phase of labor.

**Table 4:** The peanut ball positions and opinions of the participants during the labor process

		(n)	(%)
Peanut ball position in active phase	Right-left side position	36	70.6
	Half sitting position	3	5.9
	Sitting on the ball	12	23.5
Peanut ball position in transition phase	Right-left side position	15	29.4
	Half sitting position	2	3.9
	Ball pinch position	3	5.9
	Ball sitting position	21	41.2
	Forward Bending Position with Ball	10	19.6
Peanut ball position in stage 2	Half sitting position	2	4.3
	Ball pinch position	2	4.3
	Ball sitting position	20	43.5
	Forward Bending Position with Ball	22	47.8
The most prefer peanut ball position	Right-left side position	23	45.1
	Sitting on the ball	19	37.3
	Forward Bending Position with Ball	9	17.6
Would you recommend the Peanut top to other women?	Yes	51	100
	No	-	-
Would you prefer peanut balls for your next birth?	Yes	50	98
	No	1	2

Data are expressed as count (%)

## Discussion

Peanut ball application reduced labor pain and increased physical and psychospiritual comfort at birth in this study but did not affect the duration of labor (*p* < 0.05). Similarly, a study conducted by Suksesty (2017) showed that birthing balls had a significant effect on dilatation [15]. Birth is multifaceted, and this variation might arise from the effect of pelvic size and shape on position change during labor. This means that mobility supports the fetal head to achieve the optimum position and to facilitate its descent during the I stage.

Previous studies have shown that pregnant women who use a birthing ball have significantly less pain than pregnant women who do not use a birthing ball in determining the effect of a birthing ball on labor pain [16–18]. Aifa et al. (2022) stated that the use of a birthing ball in the first stage of labor significantly reduces labor pain [19]. A study conducted by Jayasudha et al. (2021) in India reported that peanut balls reduced the perception of pain [20]. Similarly, in a randomized controlled study conducted in Iran by Shirazi et al. (2019), it was reported that pregnant women who exercised with a birthing ball during labor experienced less labor pain and had greater self-efficacy for birth than those who did not [10]. Peanut ball use helps labor progress by relieving labor pain and reducing the discomfort of contractions, which indirectly reduces anxiety and increases positive birth experiences for mothers. In another study conducted in Indonesia using peanut balls, the severity of labor pain in the active phase of pregnant women using peanut balls was found to be significantly lower than that in the group that did not use peanut balls. It has also been reported that fetal head descent is faster in the group in which the peanut ball is applied [21]. In a meta-analysis conducted by Delgado et al. (2022), it was determined that pain decreased at 20, 60, and 90 min after the ball was applied [22]. Similarly, the literature and study findings indicate that the use of peanut balls during the birth process reduces labor pain. In line with the findings obtained from this study and the studies conducted, it is thought that the peanut ball provides freedom of movement to pregnant women during birth, increases self-efficacy, reduces anxiety, and reduces labor pain by increasing self-confidence.

When the duration of labor was compared in the study, the mean duration of childbirth did not differ between the groups. Although statistical significance was not obtained for variables such as labor time, possible clinical significance should not be ignored. Studies conducted with peanut balls have shown that the duration of labor is reduced [9, 11, 12, 22, 23]. This study is similar to previous studies, and unlike previous studies, no significant decrease was found in the duration of labor of the peanut ball [24, 25]. Exercise with a birthing ball improves pelvic rotation and increases the pelvic mobility of pregnant women [26]. It has been reported in the literature that the use of peanut balls reduces the duration of labor and cesarean delivery rates [9, 24, 27, 28]. In this study, cervical dilatation and effacement progressed, but although the length of childbirth was shorter in the PG, the lack of a significant difference between the groups is thought to be related to the limited number of samples.

The concepts of comfort and convenience are used similarly in the literature, and there are studies expressing comfort regarding birthing balls and peanut balls. King and Pinger (2014) state that ambulation and frequent position changes should be encouraged during birth to increase maternal comfort and optimal fetal position [29]. In this study, there was no statistically significant difference in the total scores of the groups on the birth comfort scale. The reason for this similarity is that environmental comfort, which is the birth comfort scale subdimension, is at a higher level in the control group. The positive scores of the PG increased in the physical comfort and psychospiritual comfort dimensions, and the application of peanut balls increased physical and psychospiritual comfort. Women’s satisfaction and comfort levels increase when they are supported during birth and can move freely. In studies where the peanut ball was applied, it was determined that the participants’ opinions about the peanut ball were that the peanut ball provided comfort and that their birth comfort scores were higher. Studies support that emotional support is more important than physical support. In this study, the psychospiritual comfort score was found to be high. The quality of the midwife’s relationship with the pregnant woman and her decision-making status is extremely important for the birth environment, pain, immobility, medical interventions, and continuous care support [17, 30].

In this study, it was determined that the peanut ball is an important tool for coping with labor pain and relaxing pregnant women during labor. By focusing on the balls, women made the birthing process more acceptable and their relationship with the midwife more positive. It is thought that the pregnant women in the control group received one-on-one midwife support, thus increasing the comfort level of both groups. A total of 98% of mothers stated that they wanted to use peanut balls in their future births. As a result, birth satisfaction and comfort increase by reducing birth pain.

### Limitations of the research

One limitation of the study could be the relatively small sample size, which might have impacted the statistical power to detect significant differences, particularly in outcomes such as duration of labor. The study may have taken place in a specific setting with particular practices and protocols that might not be generalizable to all birthing environments. The study may not have followed up with participants post-birth to assess any long-term effects or implications of using peanut balls during labor.

## Conclusion

This study revealed that using a peanut ball reduced labor pain, increased birth comfort, and was a tool that positively affected the birth process by helping labor progress. The peanut ball was a used midwifery intervention that was simple to use, low cost, and supported vaginal birth.

Pregnant women were provided with appropriate support and an appropriate environment to change their position, and it was observed that one-on-one midwife support increased maternal satisfaction. Training should be organized to encourage and disseminate the use of peanut balls to improve the progress of labor, reduce pain, increase comfort, and obtain a more satisfactory birth experience in hospitals and especially in pregnancy schools. More clinical studies are needed to evaluate the effectiveness of peanut balls.

### Clinical implications

Healthcare providers should educate pregnant women about the potential benefits of using peanut balls during labor to manage pain and improve comfort. Hospitals and birthing centers could consider implementing standardized protocols for the use of peanut balls during labor to ensure consistency in care. Collaboration between midwives, nurses, and other healthcare professionals is essential to provide comprehensive care and support for women using peanut balls during labor. More research is needed to explore the long-term effects of peanut ball use during labor, including its impact on postpartum recovery and maternal satisfaction.

## Supplementary Information

Supplementary Material 1

Supplementary Material 2

## Data Availability

The datasets generated and/or analyzed during the current study are not publicly available due to privacy considerations but are available from the corresponding author upon reasonable request.
