# Supplementary material for: Randomized controlled trial on the effect of the peanut ball devices on the duration of active labor, labor pain and comfort
Source: BMC Pregnancy Childbirth. 2026 Feb 17;26:322. doi: 10.1186/s12884-026-08720-2 (PMC13015091; doi:10.1186/s12884-026-08720-2)
Supplement: Supplementary file 1 — Supplementary Material 1 [file 12884_2026_8720_MOESM1_ESM.pdf]

**Birth Comfort Scale**

We would like to thank you for your participation in this study, which we aim to develop in order to diagnose the level of comfort in childbirth. For each statement below, there are five options ranging from "**strongly agree**" to "**strongly disagree**". Please circle the number that best describes your current comfort level.

| Completely I agree. | | | Absolutely . disagree | |
|---|---|---|---|---|
| 5 | 4 | 3 | 2 | 1 |

| | | | | | | |
|---|---|---|---|---|---|---|
| 1. | In my neighborhood individuals thanks to myself feeling strong | 5 | 4 | 3 | 2 | 1 |
| 2. | I work in harmony with my body. | 5 | 4 | 3 | 2 | 1 |
| 3. | More more pain I can bear it, because the pains are helping my baby to be born | 5 | 4 | 3 | 2 | 1 |
| 4. | I'm confident, I can have my baby. | 5 | 4 | 3 | 2 | 1 |
| 5. | This room makes me feel helpless. | 5 | 4 | 3 | 2 | 1 |
| 6. | Labor pains me to be strong. | 5 | 4 | 3 | 2 | 1 |
| 7. | I feel like giving up | 5 | 4 | 3 | 2 | 1 |
| 8. | I worry that I will lose control | 5 | 4 | 3 | 2 | 1 |
| 9. | This is a very safe place | 5 | 4 | 3 | 2 | 1 |
